# The Development of a Web-Based Program to Reduce Dietary Salt Intake in Schoolchildren: Study Protocol

**DOI:** 10.2196/resprot.7597

**Published:** 2017-05-31

**Authors:** Carley Ann Grimes, Alison Booth, Durreajam Khokhar, Madeline West, Claire Margerison, Karen Campbell, Caryl Nowson

**Affiliations:** ^1^ Institute for Physical Activity and Nutrition Research Deakin University Geelong Australia

**Keywords:** sodium, dietary, sodium chloride, dietary, child, Internet, nutrition, website development, Australia

## Abstract

**Background:**

Salt intake of schoolchildren in the Australian state of Victoria is high. To protect future cardiovascular health, interventions that seek to reduce the amount of salt in children’s diets are required.

**Objective:**

We sought to develop and pilot test a Web-based program (Digital Education to Limit Salt Intake in the Home [DELISH]) that aims to reduce dietary salt intake among schoolchildren and to improve child and parent knowledge, attitudes, and behaviors related to salt intake. This paper presents the DELISH study protocol, along with pilot findings used to inform the development of the program.

**Methods:**

The DELISH program is a 5-week Web-based intervention that targets schoolchildren aged 7-10 years and their parents. This is a single-arm study with a pretest and posttest design. We will assess change in salt intake through analysis of 24-hour urinary sodium excretion. Children and parents will complete online surveys assessing knowledge, attitudes, and behaviors related to salt intake. We will assess feasibility of the program via process measures, which include metrics to describe intervention uptake (eg, number of children who complete Web-based sessions and of parents who view online newsletters) and evaluation surveys and interviews conducted with children, parents, and schoolteachers. The first 2 Web sessions developed for children were pilot tested in 19 children aged 8-12 years.

**Results:**

Findings from pilot testing indicated that most children (session 1: 18/19, 95%; and session 2: 19/19, 100%) enjoyed completing each session and liked the inclusion of comic strips and interactive games. Commonly reported areas of improvement related to sessions being text and content heavy. Based on these findings, we simplified sessions and developed 3 additional sessions for use in the DELISH program. The DELISH program was implemented during June-December 2016. We expect to have results from this study at the end of 2017.

**Conclusions:**

To our knowledge, this is the first Australian study to examine the effectiveness of a Web-based program to reduce salt intake among children in primary school. If shown to be acceptable and effective in lowering salt intake, the DELISH program could be tested using a more rigorous randomized controlled trial design.

## Introduction

In adults, excess salt intake is associated with high blood pressure [1], which is a major risk factor for cardiovascular disease [2]. It has been predicted that lowering population salt intake would substantially reduce morbidity, mortality, and public health care costs [3,4]. As part of the global action plan to reduce noncommunicable diseases, the World Health Organization has recommended a 30% reduction in global salt intake by 2025 [5].

As in adulthood, childhood dietary salt intake is positively associated with blood pressure [1,6]. Findings from a meta-analysis of 10 controlled salt-reduction trials found that a 42% reduction in salt intake predicted a significant reduction in both systolic (–1.17 mm Hg) and diastolic (–1.29 mm Hg) blood pressure [7]. Although the magnitude of the association between salt reduction and blood pressure in children is relatively modest, blood pressure follows a tracking pattern from childhood into adulthood [8]; therefore, the public health benefits of shifting the distribution of population blood pressure levels is important [9]. Additional consequences of excess salt intake during early life include increased risk of obesity [10,11] and the development of taste preferences for saltier foods [12,13].

The most recent (2011-2012) Australian national estimates of sodium intake, determined via 24-hour dietary recall, showed that average intakes were 2058 mg/day (salt equivalent 5.2 g/day) among 4- to 8-year-old children and 2462 mg/day (salt 6.3 g/day) among 9- to 13-year-old children [14]. This is in excess of dietary recommendations, which specify daily intakes of no more than 1400 mg/day (salt equivalent 3.5 g/day) and 2000 mg/day (salt 5 g/day) for each respective age group [15]. Using the reference standard measure of 24-hour urinary sodium excretion, we have previously reported an average salt intake of 6.1 g/day among 4- to 12-year-old schoolchildren in Victoria, Australia, with 72% of children exceeding the age-specific guideline for salt intake [16]. These findings demonstrate the need for strategies that seek to reduce salt intake among Australian schoolchildren.

Past evidence indicated that educational messages targeting the key sources of salt in the diet [17] and behavioral skills, such as reading food labels to select lower-salt foods [18] or using herbs and spices instead of salt to flavor food [19], are key components of effective programs to reduce salt intake in adults. However, there is little evidence of effective behavioral-based strategies for reducing salt intake in schoolchildren. The expansion of Internet technologies within the home in Australia [20] provides an important opportunity to deliver public health interventions. In 2014-2015, 97% of Australian households with children aged under 15 years had access to the Internet [20]. Although there is a social gradient for Internet access in Australia, access among more marginalized groups is still relatively common (eg, 77% of those educated to grade 12 or below have Internet access) [20]. Research suggests that online technologies can be used to engage children to achieve positive changes in dietary behaviors [21,22], for example, improved intakes of fruit and vegetables [23]. To date, no Web-based behavioral intervention targeting salt reduction in Australian schoolchildren has been trialed. Hence, we sought to develop and pilot test a Web-based salt-reduction program that targets primary school children and their parents. The primary objective of the program is to reduce salt intake among children by 20% and improve child and parent knowledge, attitudes, and behaviors related to salt intake. Secondary objectives relate to taste preferences in children; these include (1) assessing their salt preference for commercially available foods of varying salt concentration, (2) assessing the association between salt preference and salt intake, and (3) assessing whether participation in the program alters salt preference.

The purpose of this paper is to describe the Digital Education to Limit Salt Intake in the Home (DELISH) program and data collection methods that we will use in the study. In addition, we report on findings related to the development of the program, which includes pilot testing of educational materials among a sample of children aged 8-12 years.

## Methods

### Study Design and Participants

The DELISH program is a 5-week Web-based intervention that targets schoolchildren in years 2-4 of schooling (ages 7-10 years) and their parents. This is a single-arm study with a pretest and posttest design. The age range of participants is restricted to facilitate the design of appropriate intervention materials with respect to reading ability and comprehension. Children and parents are targeted, as research suggests that interventions that target the whole family, as opposed to only the parent or child, are more effective for achieving dietary changes [24]. Ethics approval has been granted by the Deakin University Health Human Ethics Advisory Group (Project No. HEAG-H 37/2016) and the Department of Education and Training, Victoria State Government (2015_002884). We will obtain written informed consent from the primary caregiver and assent from the child before their participation. This study is supported by a Heart Foundation Vanguard grant (Application ID: 100574).

### Recruitment Procedures

We will recruit children from government primary schools located in the Greater Geelong Region of Victoria, Australia. We will use a Web-based school locater search engine to identify schools with enrollments for primary school children [25]. The school’s postcode and corresponding Index of Relative Socio-economic Advantage and Disadvantage [26] will be used to group each school into low-, medium-, and high-socioeconomic areas of Victoria. Following this, we will randomly select schools from each tertile and invite each school’s principal to participate in the study via an email invitation and a follow-up courtesy call reminder. Classroom teachers will be provided with information about the study. Prior to school participation, written consent will be obtained from the school principal and classroom teachers. Following this, children in school grades 2-4 (7-10 years of age) will receive a study information pack, which will include an invitation letter addressed to parents, and a parental and child plain language brochure and consent forms. We will also advertise the study through the schools’ newsletters.

### Inclusion Criteria

Inclusion criteria are that children (1) will be required to have a parent with an email address and have access to a computer or tablet at home with an Internet connection, and (2) must be attending a Victorian government primary school in years (ie, grade) 2-4.

### Intervention Overview

The Web-based program will be delivered over a 5-week period. Children will actively participate in weekly Web-based sessions designed to take approximately 20-30 minutes each to complete. Parents will receive concurrent educational materials through weekly online newsletters sent via email and short message service (SMS) text messages. In addition, there will be a central study website, where resources for children and parents will be posted.

### Sample Size Calculations

We designed the intervention to reduce salt intake by 20% (ie, 1.2 g/day of salt). Using a random intercept multilevel model (with salt intake outcome allowed to vary randomly at both the individual and the school level) to estimate the change in salt intake over time (pre vs post as a fixed effect) and assuming a mean baseline salt intake of 6.0 (SD 2.5) g/day and intraclass coefficient of 0.04 (school clusters), we require a sample size of 102 children to detect a difference of 1.2 g/day of salt with 90% power at *P*<.05. Anticipating a 20% rate of dropout or incomplete urine samples returned, we aim to recruit 122 children across 6 schools.

### Intervention Development

#### Behavioral Objectives of the Intervention

We developed 3 key behavioral messages to reduce salt intake in 7- to 10-year-olds: (1) *stop* using the salt shaker during cooking and at the table, (2) *switch* to lower-salt foods by checking food labels (focus on bread, breakfast cereal, and cheese), and (3) *swap* processed salty foods (eg, processed meats, take-out pizzas and burgers, savory sauces, and snack foods) with healthier alternatives.

We assessed the potential effectiveness of these strategies to meet the primary outcome of a 20% reduction in salt intake via dietary modelling and deemed them as suitable (Multimedia Appendix 1 [27-31]). The rationale for the development of these messages is described below.

The selection of food groups to target within the intervention was informed by our previous work, which identified the main dietary sources of salt among Australian children [32]. These are bread (15%); processed meat (9%); savory sauces and condiments (6%); mixed cereal dishes (7%), which include pizza, sandwiches, and hamburgers; cheese (5%); breakfast cereals (4%); pastries (4%), which include meat pies and sausage rolls; and snack foods (4%). As sodium is widespread within the food supply [33], the main sources of dietary salt are diverse in their nutritional composition and include discretionary options, which should be limited in the diet (eg, processed meats and pastries), as well as core foods, which should form the basis of a healthy dietary pattern (eg, bread and cheese) [34]. We have previously reported that core foods provided more than half of all salt consumed (55%) by schoolchildren, with the remaining 45% provided by discretionary foods (unpublished data, 2017). Hence, to reduce salt intake, there is a need for strategies that incorporate messages related to overall healthy eating principles, such as limiting the consumption of discretionary foods, as well as messages related to the consumption of core foods that have lower levels of salt. The large variability of salt content within core foods [33,35] makes finding lower-salt options feasible. On this background, we used the *Australian Guide to Healthy Eating* (AGTHE) as an evidence-based healthy eating tool to embed salt-specific messages in the intervention. The AGTHE is a food selection guide that groups foods as core or discretionary choices and provides a visual representation of the proportion of the 5 food groups recommended for consumption each day [34].

We also considered the contribution of discretionary salt use to intake. While no data for Australian children are available, in Western countries it is estimated that approximately 6% of sodium consumed comes from salt added at the table and 5% is added during cooking [30,36]. Previously, we have shown in Victorian schoolchildren that reported table salt use was related to overall higher salt intake, as measured by 24-hour urinary sodium excretion [37]. In addition, we have previously reported that the use of table and cooking salt is relatively common among Victorian schoolchildren and their parents (eg, 40% of children reported using table salt and 66% of parents reported using cooking salt) [31]. These findings demonstrate the importance of targeting discretionary salt use.

Finally, to aid in the development of tailored strategies, we considered the contribution of salt from different meals as consumed by Australian children participating in the 2007 Australian National Children’s Nutrition and Physical Activity Survey [27]. We found that foods consumed at dinner contributed the most to daily salt intake (35%) (Multimedia Appendix 2 [27]). Although foods consumed during lunch provided less salt overall (25% of daily intake), these foods had the highest sodium density, indicating the consumption of particularly salty foods at lunch. These findings indicate the importance of targeting lunchtime and dinnertime food intake to help reduce daily salt intake.

#### Theoretical Framework

Social cognitive theory is frequently used in dietary interventions targeting children [24], and it informed the development of this intervention. Social cognitive theory stipulates that behavior is determined by the reciprocal interaction of personal cognitive factors (eg, self-efficacy, outcome expectations, and knowledge), socioenvironmental factors (eg, observational learning, normative beliefs, social support, barriers, and opportunities), and behavioral factors (eg, behavioral capability, intentions, and reinforcement) [38].Developing strategies that address the constructs within social cognitive theory increases the likelihood of influencing behavior change [38]. In particular, we focused on those constructs previously shown to be related to dietary intake in children. These include self-efficacy [39], intentions (ie, goal setting) [40,41], reinforcements [42], and knowledge [43]. We developed strategies to address the intervention content with reference to Michie and colleagues’ [44] taxonomy of behavior change techniques. We selected behavior change techniques for inclusion in the intervention considering what was appropriate given the intervention mode of delivery (ie, Web based, with no face-to-face contact) and what behavior change techniques have previously been used in effective interventions targeting children’s eating behaviors [24,45]. In addition, we considered previous reports of what behavioral-based strategies were effective in reducing salt intakes. For example, in adults and children, effective strategies to reduce salt intake include providing education on reading sodium information included on food labels, cooking recipes with spices and herbs, information on selecting lower-sodium foods when eating out, and goal setting [18,19,46,47]. Multimedia Appendix 3 shows a list of strategies included in the intervention, mapped to behavior change techniques and social cognitive theory constructs.

#### Intervention Content

Storytelling can be used as a means to communicate health messages and facilitate learning outcomes in children [48,49]. In conjunction with a creative writer and illustrator (Ben Pearmain Illustration, Melbourne, Australia) we created a narrative within which to embed the intervention content. To help engage children, we selected a detective theme as a basis of the story, with a personified dog as the protagonist [49,50]. Comic strips will deliver the story at the beginning of each Web session (Multimedia Appendix 4), which will set the scene for the weekly learning activities for the children to complete.

#### DELISH Starter Pack

Following the collection of baseline data, we will mail a starter pack to the family home. The pack will include a 1-page overview of the program; a fridge magnet outlining the 3 key behavioral objectives of the program; AGTHE educational resources (including fridge magnet and pamphlets); a cheat sheet shopping card outlining sodium content targets for bread, breakfast cereal, and cheese; a detective logbook for the child to record weekly goals and stick badges in; and a “stop using the salt shaker” sticker and stickers of badges for completing case files and meeting goals.

#### Web-Based Sessions

**Figure 1:**
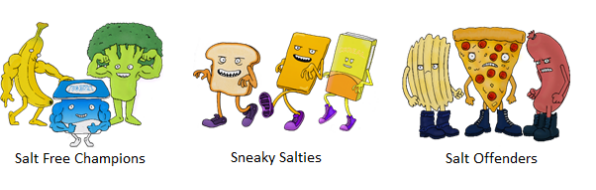
Characters used to convey key messages to children.

**Figure 2:**
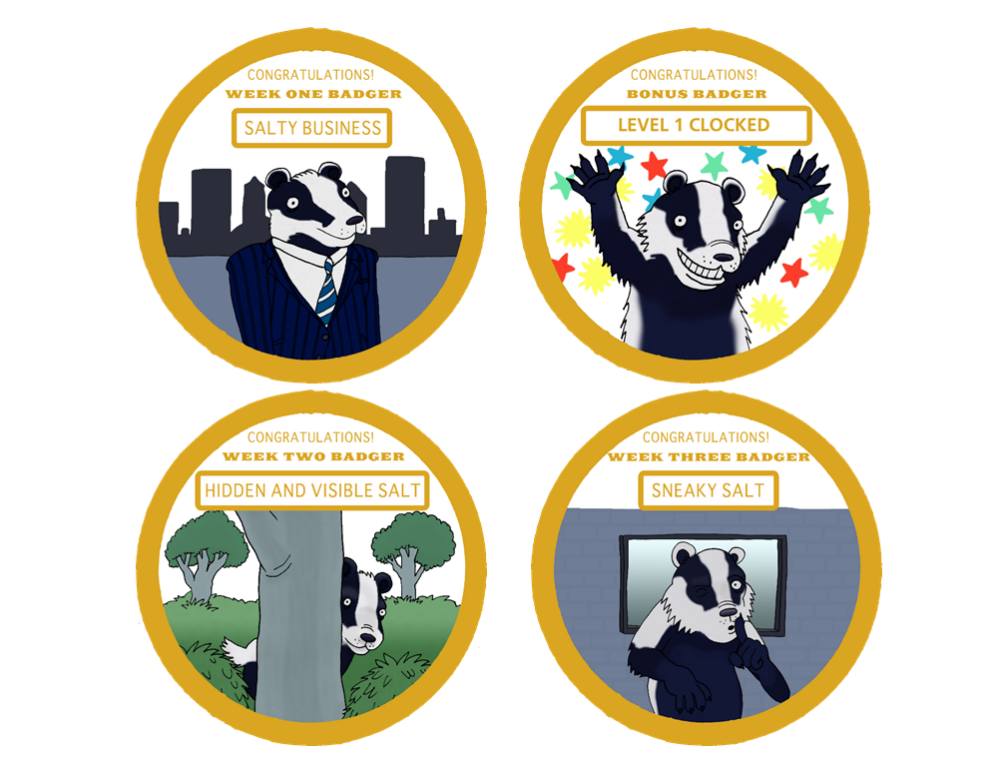
Badges provided for completing Web-based sessions and meeting goals.

Parents will receive a weekly email with access to the Web-based session; alternatively, children will be able to access each session via the study website. We developed the Web sessions using the e-learning software Articulate Storyline 2 (Articulate Global, Inc). To facilitate engagement and learning, the sessions will be packaged as a series of detective case files for the child to solve. Each case file targets key learning objectives related to the behavioral messages of the intervention (outlined in detail in Multimedia Appendix 3). Table 1 provides an overview of each session. Sessions we developed are interactive and include comic strips, characters to introduce key concepts (Figure 1), activities and games, video content, and sound effects. Multimedia Appendix 4 presents the basic format of the sessions. If the child completes the session and solves the case file, a badge is awarded (Figure 2).

**Table 1:** Overview of weekly Web-based sessions for children.

Week	Session name	Session overview
1	Salty Business	Background narrative is introduced, child signs up to detective program, and program structure is outlined. Key information related to salt is presented: difference between salt and sodium, the function of salt in the body, the link between excess salt and health, and dietary recommendations for salt.
2	Hidden & Visible Salt	Dietary sources of salt, including processed foods and discretionary salt, are outlined. Concept that fresh, unprocessed foods do not contain added salt is introduced (ie, “Salt Free Champions”). Key message 1, “Stop using the salt shaker,” is introduced.
3	Sneaky Salt	AGTHE^a^ is introduced, as well as the concept that some core foods contain added salt (ie, “Sneaky Salties”). Information on reading food labels to find foods with less salt is provided. Key message 2, “Switch to lower salt foods by checking food labels,” is introduced.
4	Salt Swaps	AGTHE is used to provide information on discretionary foods and “Salt Offenders” are introduced. Key message 3, “Swap processed salty foods with healthier alternatives,” is introduced.
5	Wrap-up session	The story concludes, and the 3 key messages to reduce salt intake (ie, Stop, Switch, and Swap) are reiterated. As child has solved all 4 case files, they are promoted to a chief investigator.

^a^AGTHE: *Australian Guide to Healthy Eating*.

#### Goal Setting

At the end of each session (weeks 2, 3, and 4), the child will be asked to set a goal that is related to the key behavioral message for that week. Given the ages of the children, we created predefined options for goals from which the child could select [51]. We will provide information on possible barriers to meeting the goal and solutions to overcome. Children will be encouraged to record their goal in the detective logbook and discuss this with their parent. If the child reports meeting their goal in the following week, they will be awarded a bonus badge (Figure 2).

#### Pilot Testing of Web-Based Sessions

The first 2 Web sessions that were developed were pilot tested in children aged 8 to 12 years. We recruited children via advertisements and email invitations that we distributed among networks at our workplace (ie, Deakin University). Parent and child consent was provided and ethical approval was granted by the Deakin University Health Human Ethics Advisory Group (Project No. HEAG-H 142_2014). Parents were emailed hyperlinks for their child to access the 2 Web-based sessions. At the end of each Web session, a hyperlink was provided to access an evaluation survey to determine the acceptability and appeal of each session. We invited a subsample of children to participate in a face-to-face interview to provide more detailed feedback on each session. The results section below presents findings from this evaluation.

#### Parent Newsletters

**Figure 3:**
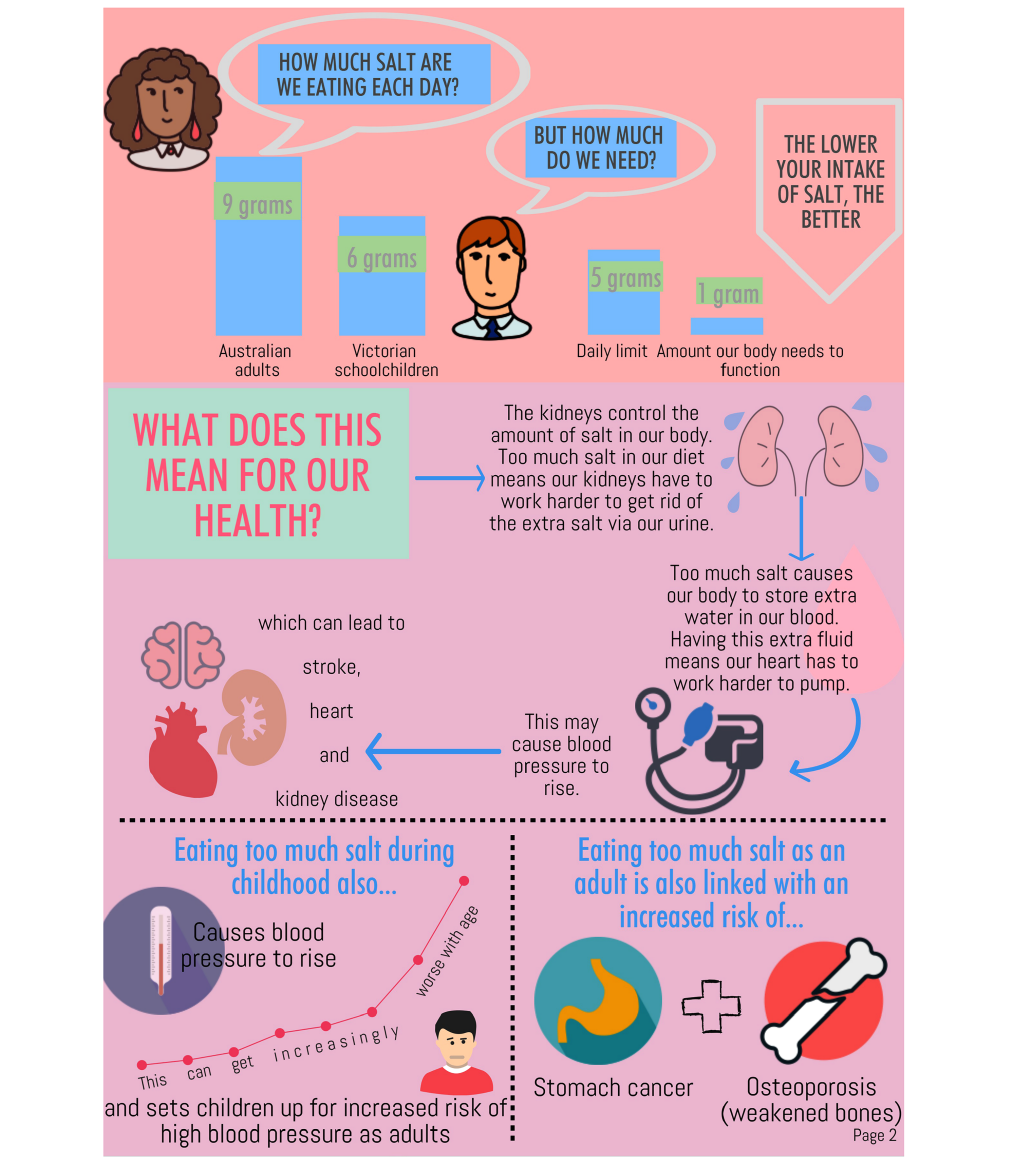
Example from online newsletter for parents.

Parents will receive weekly emails with hyperlinks to access the online newsletter. The newsletters include educational materials relevant to the week of the program and information on the child’s weekly goal (Figure 3). Additional hyperlinks will be embedded within newsletters directing parents to extra resources, such as a video for reading food labels, supermarket cheat sheets for top picks for foods with less salt, and an herbs and spice resource for cooking without salt. The content of newsletters has been reviewed by a dietitian and tested with 2 mothers, and where necessary changes were made to the language, layout, and graphics.

#### Study Website

**Figure 4:**
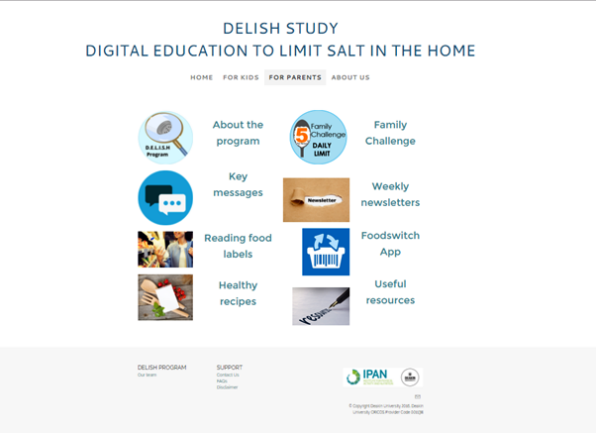
Parents' section of the study website.

A password-protected study website will be accessible during the intervention and will provide resources for children and parents. We will update the website weekly with relevant resources. The children’s section will include access to the detective case files (ie, Web-based sessions), downloadable versions of the comics, weekly case badges, bonus badges, and handouts describing potential barriers to meeting weekly goals with suggested solutions. The parent’s section will contain access to online newsletters, also available to download in PDF format, as well as other key resources (Figure 4). In relation to recipe resources, we used existing recipes from health agencies, such as the National Heart Foundation of Australia and World Action on Salt & Health, and where appropriate we provide information for modifying the recipe to reduce salt.

#### SMS Text Messages

We will send 2 to 3 reminder SMS text messages to the parent during each week of the intervention. The messages will provide information related to weekly key messages and goal setting, and will include hyperlinks to access intervention resources, such as online newsletters and materials posted on the website.

### Data Collection Methods

Table 2 provides an overview of measures to be completed before and after the intervention.

#### Urine Collection

The primary outcome of the DELISH program is the change in the child’s salt intake assessed via a 24-hour urine collection. To engage parents, we will provide them with the option of completing a 24-hour urine collection; however, this will not be mandatory. We will follow our previous protocol for collecting 24-hour urine samples in schoolchildren [52]. Briefly, the first morning urine will be voided, and this will be recorded as the 24-hour urine start time. Following this, all urine will be collected, finishing with a final collection at the corresponding 24-hour finish time. Participants will be able to commence the 24-hour urine collection either at school or at home over the weekend. We will instruct participants to complete the postintervention 24-hour urine collection on the same type of day (ie, school or nonschool day) as the baseline assessment. Children will be provided with a 2.5-L widemouthed, rimmed polypropylene bottle to collect urine. To assist with urine collection, we will provide an additional 500-mL plastic handled jug. Verbal and written instructions will be provided. Children will be asked to report any missed collections or spillages on a urine collection slip, which will be returned with the 24-hour urine sample. We will standardize urine volume and electrolytes to a 24-hour period; for example, [24 hours/urine duration (hours)] × urinary measure. We will consider 24-hour urine samples to be complete if no more than 1 collection is reported as missing (provided that we find no differences in urinary sodium by sex between those who report 1 missed collection and those who report no missed collections), duration of collection is within 18-34 hours, total volume is 300 mL or more, and urinary creatinine excretion (mmol/kg body weight/day) is greater than the fifth percentile cutoff point obtained from previous reference data among Victorian schoolchildren (ie, 7-8 years, ≥0.1 mmol/kg body weight/day; and 9-10 years, ≥0.1 mmol/kg body weight/day [52]).

The first morning void of the 24-hour urine collection will be collected in a separate container, before the start of the 24-hour collection, and will be assessed for sodium concentration [53]. Research staff will visit the school on the day preceding the scheduled testing to provide those children completing a school-day 24-hour urine collection with a 500-mL plastic collection container and carry bag, along with verbal and written instructions, so the child can collect the overnight urine sample on waking on the day before starting the 24-hour urine sample at school. Children who complete weekend-day 24-hour urine collections will be provided with the collection materials and instructions for both the 24-hour urine collection and overnight collection on the day of testing at the school. Children will be asked to report onto a urine collection slip any spillages while collecting the overnight urine.

Urine samples will be transported to a commercial pathology company for urinalysis. Total volume of both urine samples will be measured, and each will be assessed for sodium and potassium concentrations using indirect ion-selective electrodes and for urinary creatinine concentration using the Jaffe reaction [54] on the Siemens Advia 2400 analyzer (Siemens Healthcare GmbH, Erlangen, Germany). Per participant, 2×10-mL aliquots will be taken from the 24-hour urine collection for storage and transferred to –80°C conditions. We will use the molecular weights of sodium (23 g/mol), sodium chloride (58.5 g/mol), and potassium (39.1 g/mol) to convert laboratory values of millimoles to milligrams [55].

#### Anthropometry (Child)

Trained research staff will collect anthropometric measurements at the school site on the day of testing. Height will be measured to the nearest 0.1 cm using a calibrated portable stadiometer, seca model 220 (Hamburg, Germany). Weight will be measured to the nearest 0.1 kg using a calibrated UC-321 portable electronic scale (A&D Medical, San Jose, CA, USA). Waist circumference will be measured to the nearest 0.1 cm using a Lufkin Executive Thinline W606PM pocket tape (Sparks, MD, USA). Waist circumference will be measured at the end of a normal expiration at the narrowest point between the lower costal border and the top of the iliac crest. For all anthropometric measures, 2 measurements will be taken. If these 2 measures differ by more than 5 mm for height, 0.1 kg for weight, or 10 mm for waist circumference, a third measurement will be taken. Where 2 measurements are taken we will use the mean in the analysis, and where 3 measurements are taken we will use the median value. We will calculate body mass index as body weight (kg) divided by the square of body height (m^2^). Age- and sex-adjusted body mass index *z* scores will be calculated using the LMS method [56] with the 2000 US Centers for Disease Control and Prevention growth charts acting as the reference population [57]. Participants will be grouped into weight categories (very underweight, underweight, healthy weight, overweight, obese) using the age- and sex-specific International Obesity Task Force body mass index reference cutoffs for children [58,59].

#### Dietary intake (Child)

To assess the dietary intake of children at baseline, the parent will complete an online version of the validated Australian Child and Adolescent Eating Survey (ACAES) [60]. The ACAES is a 120-item, semiquantitative food frequency questionnaire that includes a comprehensive list of foods, enabling the estimation of all macronutrients and key micronutrients. We will compute the nutrient data from the ACAES using FoodWorks (version 3.02.581; Xyris Software [Australia] Pty Ltd). Parents will be instructed to report on their child’s dietary intake for the previous month. The purpose of collecting information from the ACAES is to provide background information on overall dietary intake within the group.

#### Online Salt Survey (Child)

We developed a 29-item online survey assessing the constructs of knowledge (20 questions), attitudes (2 questions), behaviors (4 questions), and self-efficacy (3 questions) related to dietary salt. To simplify the survey and help with readability, we incorporated images into response options. The survey instrument was pilot tested with 3 schoolchildren aged 7-9 years, 2 mothers, 1 primary school teacher, and 2 dietitians to check for readability, age appropriateness, and content. Following this testing, we made small modifications to the survey. An online readability tool graded the survey instrument as “easy to read” and appropriate for 8- to 9-year-olds [61]. We will assess test-retest reliability of the questionnaire in a separate sample of 45 schoolchildren.

The knowledge questions include declarative (eg, food sources of salt, relationship between salt) and procedural knowledge (eg, reading food labels to pick foods with less salt). Response scales include multiple choice options, along with “true,” “false,” and “not sure” responses, with the latter included to discourage guessing. Correct answers will be scored 1 point and incorrect answers, including “not sure,” will be scored as 0, with a total maximum knowledge score of 20. Attitude questions assess the importance of salt in food to make it tasty (eg, “Salt makes food tasty”), and these questions were modelled from a previous survey conducted in adults [62] and were simplified for use with children (eg, the response scale was modified to a 5-point Likert smiley-face scale). Responses will be scored as “agree a lot”=3 points, “agree”=2 points, “I’m not sure”=0 points, “disagree”=0 points, and “disagree a lot”=0 points; hence, higher scores will reflect stronger beliefs about the importance of salt in food for taste. Behavior questions are related to discretionary salt use, previously used in children [52], and specific behaviors targeted within the intervention, such as talking to parents about salt use at home. Behavior question response items will be scored from 0 to 3, or 0 to 1 in the case of 2-item responses, with a higher score indicating better adherence to targeted salt-related behaviors. Self-efficacy questions were based on those used in previous studies in children [63] and examined key behaviors targeted within the intervention. The 3 response item will be scored as 0 to 2 (“not sure at all,” “a little sure I can,” and “very sure I can”) indicating low, medium, and high self-efficacy, respectively.

**Table 2:** Overview of data collection procedures.

Participants			Baseline	Postintervention
**Child**				
	**Face-to-face school measures**			
		1 × 24-hour urine sample	✓	✓
		1 × overnight urine sample	✓	✓
		Weight, height, and waist circumference	✓	✓
		Taste-testing session	✓	✓
	**Online measures**			
		Australian Child and Adolescent Eating Survey (completed by parent proxy)	✓	
		Child salt survey to assess knowledge, attitudes, and behaviors	✓	
		Program evaluation questionnaire		✓
		Evaluation interview (subsample n=10)		✓
**Parent**				
		1 × 24-hour urine sample (optional)	✓	✓
		Parent salt survey to assess knowledge, attitudes, behaviors, and demographic characteristics	✓	✓
		Program evaluation questionnaire		✓
		Evaluation interview (subsample n=10)		✓
**Teachers/principals**				
		Evaluation interview		✓

#### Online Salt Survey (Parent)

The study parent will complete an online questionnaire, containing 34 questions (baseline) and 21 questions (postintervention) assessing sociodemographic characteristics such as age, sex, and education level (13 questions) and knowledge, attitudes, and behaviors related to salt intake. The survey was based on a previously validated salt knowledge questionnaire [62,64], while other questions were modelled on those used in past surveys [65-67], and was tested for readability in 5 parents from varying demographic backgrounds. The pre- and postintervention surveys for parents were identical, with the exception of excluding demographic information on the follow-up survey.

We included 14 knowledge questions assessing declarative (ie, relationship between salt and sodium, current recommendation, main sources, health conditions related to excessive salt intake) and procedural knowledge (interpreting sodium information on food labels). These question responses are in the form of multiple choice and 5-point Likert scale (“strongly disagree” to “strongly agree”). All correct responses will be scored as 1, while incorrect responses, including “don’t know” and “not sure,” will be scored as 0. Responses to Likert scale questions will be scored as a 2 for “certainly true,” 1 for “probably true,” and 0 for incorrect answers, including “not sure” and “don’t know” responses. Negative statements will be reversed prior to scoring. The salt knowledge questions will be summed to generate scores for each question, and a total salt knowledge score will be derived by summing all the knowledge questions. The minimum and maximum salt knowledge scores are 0 and 32, respectively. We added some additional questions assessing knowledge specific to salt consumption in children, as the original validated questionnaire was not directed to parents.

We assessed 3 attitude questions (ie, parents’ personal attitude toward their own salt intakes, salt as a flavor enhancer, taste of low-salt foods, and importance of reducing salt) using 5-point Likert scales (“strongly disagree” to “strongly agree”). Scores will be assigned from 1 (“strongly disagree”) to 5 (“strongly agree”). The scores will be summed to derive a total beliefs score. The minimum and maximum attitude scores will be 0 and 6, respectively, with a score of 3 assigned for “strongly agree,” 2 for “agree,” 1 for “neither agree nor disagree,” 0 for “disagree” or “strongly disagree.” Higher scores will indicate stronger beliefs about the importance of the salt in food for taste. Some questions regarding attitudes related to salt intake will be presented as percentages.

We assessed 4 behavior questions regarding discretionary salt use (ie, adding salt to food during cooking [parent] or at the table [child and parent], and placing a salt shaker on the table [child and parent]) using 5-point Likert scales. Scores will be assigned from 0 for “always” to 4 for “never.” Parents will report the frequency of current actions taken to reduce their child’s salt intake (and actions taken in the past 1 month in the follow-up survey) using a 7-point Likert scale. The scores will be summed to derive a total behaviors score according to frequency of engaging in behavior (6 for “never,” 4 for “2-3 times/week,” etc). The minimum and maximum behavior score will be 0-53, respectively. Higher scores will indicate a higher frequency in engaging in positive salt related behaviors.

#### Taste-Testing Procedures

Tastings will be conducted with 2 commercially available food products, 1 snack food (potato chips) and 1 staple food (cornflakes), both of which are important sources of dietary salt for children [32]. We used the sodium content listed on packaged nutrient information panels to select 3 products of varying salt content for each food (Table 3). Nutrient profiles, particularly sugar and fat, were matched as closely as possible between samples of the same food type, as was visual appearance. Children will complete 2 taste tests for each food type during the testing session completed at schools (1) preference tests and (2) ability to rank samples according to salt content.

**Table 3:** Sodium content of chip and cornflake samples.

Food products		Sodium (mg/100 g)	Salt equivalent (g/100 g)
**Chips**			
	No added salt	14	0.04
	Mid salt	200	0.5
	High salt	486	1.2
**Cornflakes**			
	Low salt	90	0.2
	Mid salt	390	1.0
	High salt	590	1.5

All samples of chips and cornflakes will be presented in plain containers in a predetermined randomized sequence. Children will be asked to have a drink of water between tasting each food sample. On completion of tasting the 3 samples, children will be asked which one they liked the most (ie, preference). The most liked sample will be removed from sight and the question repeated for the remaining 2 food samples. This forced-choice rank-order method means that each child will rank the 3 samples from most to least preferred. Following the same procedure, the child will then be asked which sample they think tastes the saltiest (ie, ability to rank samples). This rank-order method is based on previous methods [68] and has been successfully used in a similar sample of children ranking sour intensities and preference [69].

### Data Analysis

We will use Stata SE 14 (StataCorp LP) for all statistical analyses. Descriptive statistics will describe continuous (mean, SD) and categorical (n, %) measures. McNemar test will assess the change in proportion of responses in the pretest and posttest surveys. Multilevel regression models will assess the change in salt intake among children, as well as child and parent salt survey scores (knowledge, attitudes, and behaviors). Models will adjust for potential confounders (ie, age, sex, and parental level of education). To assess whether participation in the program alters salt preference, we will group participants based on the sample they most preferred, and we will use the McNemar test to assess the change in proportion of children’s preferences before and after the intervention. Statistical significance will be set at *P*<.05.

### Process Evaluation

To assess the feasibility and acceptability of the program, we will collect a range of process evaluation measures. To assess the extent of the intervention delivered to children and parents, we will collate metrics for use of the study website (ie, page views and unique visitors), including the number of views for each week’s online newsletter provided to parents. For the weekly Web session for children, we will record the number of unique visitors, number of views, proportion of the session completed, and duration to complete the session. The number of children who report setting weekly goals, as well as whether those goals were met will be recorded. At the end of the intervention, we will ask parents and children to complete an anonymous online evaluation questionnaire to assess the acceptability of materials and assess any barriers that may have limited engagement. Interviews will be held with a subsample of children (n=10) and the primary caregiver (n=10) to assess their overall enjoyment of the program and materials. For children, this will be a face-to-face interview completed at the child’s school. For parents, this will be a telephone interview. We will invite participating teachers and principals to a short interview to assess their thoughts on the feasibility of incorporating a salt education program within their current curriculum and about their use of existing food or nutrition-related programs within their teaching. The interviews will take place either face-to-face or over the phone. All interviews will be audio recorded, transcribed verbatim, and analyzed for themes using NVivo software version 11.3.1 (QSR International).

## Results

### Developmental Phase of Intervention: Pilot Testing of 2 Web-Based Sessions

A total of 19 children between the ages of 8 and 12 years (n=12, 63% boys) completed both of the Web-based sessions and corresponding evaluation surveys. Of these children, 5 (3 boys) completed the additional interview. Overall, the sessions were well received. Most children (session 1: 18/19, 95%; and session 2: 19/19, 100%) reported that they liked completing the sessions, as well as the inclusion of the comic strips and the dog protagonist. All children liked the interactive games (Table 4). When children were asked to list one thing they liked most about each session, the most common responses related to the dog character, being a detective, the comic strips, and the incorporation of activities (such as using a salt detector to find out what foods have salt) and games. Children also reported that they liked learning things about salt that they didn’t know. When children were asked to list one thing they did not like about each session, some children (n=4 in session 1, n=8 in session 2) reported nothing; other responses related to technical glitches, text (eg, too many words, text changing too quickly, small font size), the speed at which children had to complete some activities, and a limited selection of avatars. More than half of the children found the activities included in each session easy to complete; however, some did report having difficulties (Table 4). When asked to specify why they found any part of the activities difficult, their responses related to use of small text that progressed quickly, hence making content difficult to read, and the inclusion of too many questions to answer.

With relation to findings from the interviews, overall, children indicated that they liked completing the sessions and found the format of the information presented to be interesting and engaging. Some children indicated that there was too much information in session 1 and that they could not understand some words used. Overall, children reported that they liked the overall look and layout of the sessions, along with the detective theme and background story. Multimedia Appendix 5 provides a selection of quotes from the interviews.

Based on findings from pilot testing, the first 2 Web sessions were modified and the remaining 3 sessions were created. Modifications related to using less text to deliver content and instead using more pictures, using a larger text font, including a seek bar to enable children to replay text and access the content delivered, testing further to remove technical glitches, and slowing down and simplifying activities (eg, foods moving across a conveyor belt to test salt levels). While we noted that children wanted more variety and interesting avatars to select from, we made no changes due to limitations in access to artwork. All of the final Web sessions used in the DELISH program were reviewed for developmental appropriateness and comprehension by a primary school teacher and were further tested with 2 primary school children. Where necessary, we made minor changes to language.

### Data Collection in the Main DELISH Study

Data collection was completed in December 2016. We are now collating and cleaning the data. Analysis will be conducted in June 2017 and we expect to have results at the end of 2017.

**Table 4:** Quantitative findings from pilot testing of 2 Web-based sessions.

Question		First session		Second session	
		n	%	n	%
**Did you watch the education session until the end?**					
	Yes	19	100	19	100
	No	0	0	0	0
**Did you like completing the online education session?**					
	Didn’t like it at all	0	0	0	0
	Didn’t like it	1	5	0	0
	Don’t know	0	0	0	0
	Liked it	8	42	9	47
	Liked it very much	10	53	10	53
**Did you like the comic strip at the start of the education session?**					
	Didn’t like it at all	1	5	0	0
	Didn’t like it	1	5	1	5
	Don’t know	0	0	2	11
	Liked it	8	42	5	26
	Liked it very much	9	47	11	58
**Did you like the detective dog, Banjo, that led you through the education session?**					
	Didn’t like it at all	1	6	2	11
	Didn’t like it	2	11	0	0
	Don’t know	0	0	1	5
	Liked it	4	22	5	26
	Liked it very much	11	61	11	58
**How difficult was it to complete the activities included in the education session?**					
	Very easy	3	16	4	21
	Easy	10	53	11	57
	Difficult	3	16	2	11
	Very difficult	1	5	0	0
	Not sure	2	11	2	11
**Did you enjoy completing the quiz/game at the end of the session?**					
	Didn’t like it at all	0	0	0	0
	Didn’t like it	0	0	0	0
	Don’t know	0	0	0	0
	Liked it	13	68	4	21
	Liked it very much	6	32	15	79
**Did you find the information included within the education session interesting?**					
	Didn’t like it at all	0	0	0	0
	Didn’t like it	1	5	1	5
	Don’t know	0	0	1	5
	Liked it	8	42	7	37
	Liked it very much	10	53	10	53
**Are you looking forward to completing the 2nd education session?**					
	Not really looking forward to it at all	2	11	N/A^a^	N/A
	Not really looking forward to it	0	0	N/A	N/A
	Don’t know	1	5	N/A	N/A
	Looking forward to it a little bit	5	26	N/A	N/A
	Yes really looking forward to it	11	58	N/A	N/A

^a^N/A: not applicable.

## Discussion

Lowering exposure to dietary salt during childhood is likely to have important cardiovascular health benefits [70]. In Australia, no Web-based behavioral programs to reduce salt intake among schoolchildren have been developed or tested. The process findings related to usability and acceptability of the DELISH program by parents and children, along with teachers’ views on incorporating salt-reduction messages within school nutrition programs, can be used to refine and modify the program as necessary. We acknowledge that the study is limited by the pretest and posttest design; as such, any reported changes in outcomes could be due to other confounding factors, such as wider health-related initiatives conducted at the participating schools. If shown to be acceptable and effective in lowering salt intake, the DELISH program could be tested using a more rigorous randomized controlled trial design.

## Appendix

Multimedia Appendix 1 Dietary modelling to assess impact of intervention strategies on sodium (ie, salt) intake.

Multimedia Appendix 2 Sodium intake in Australian schoolchildren aged 6-16 years by eating occasion (n=2921).

Multimedia Appendix 3 Overview of intervention objectives, content, and strategies mapped to behavior change techniques and social cognitive theory construct.

Multimedia Appendix 4 Overview of format for weekly Web-based sessions for children.

Multimedia Appendix 5 Selection of quotes from interviews during pilot testing of 2 Web sessions.

## Abbreviations

ACAES: Australian Child and Adolescent Eating Survey
AGTHE: Australian Guide to Healthy Eating
DELISH: Digital Education to Limit Salt Intake in the Home
SMS: short message service
