# Supplementary material for: The Development of a Web-Based Program to Reduce Dietary Salt Intake in Schoolchildren: Study Protocol
Source: JMIR Res Protoc. 2017 May 31;6(5):e103. doi: 10.2196/resprot.7597 (PMC5471360; doi:10.2196/resprot.7597)
Supplement: Multimedia Appendix 1 [file resprot_v6i5e103_app1.pdf]

Multimedia appendix 1. Dietary modelling to assess impact of intervention strategies on sodium (i.e. salt) intake [a]

Food group	Average sodium content mg/100g[b]	Target sodium content mg/100g[c]	% reduction in sodium if target met	Average sodium intake from each food group (mg)[d]	Sodium reduction (mg)
Bread	480	400	16	329	53
Breakfast cereal	421	400	5	107	5
Cheese	748	660	12	108	13
Sausages	757	80	89	76	67
Processed meat	1404	80	94	187	175
Savoury sauces	1021	500	51	96	49
Table salt reduction[e]					143
**Total Sodium reduction**					**505**[f]

[a] Based on 1 day of 24-hr dietary recall data collected in the 2007 Children's Nutrition and Physical Activity Survey [27].
[b] Based on Australian food products consumed by 4-13 year olds in the 2007 Children's Nutrition and Physical Activity Survey
[c] A target for the sodium content for each food group was set. For those 'processed' food groups targeted by the message 'swap to a healthier alternative' the target for salt corresponded to the mean sodium content of muscle meat (unprocessed) products in food composition database AUSNUT2007 [28]. For food groups targeted by the message 'switch to lower salt foods by checking food labels' the target corresponded to the Australian Division of World Action on Salt and Health target for sodium levels in Australian foods [29].
[d] Current average sodium intake from each subcategory in 4-13 year olds in the 2007 Children's Nutrition and Physical Activity Survey
[e] Table salt use accounts for 6% of intake [30]. Based on previous 24-hr urinary Na excretion data among 4-12 year old Victorian schoolchildren, sodium intake was 103 mmol/d [31]. 0.06*103= 6.2 mmol/d = 143 mg/d
[f] Sodium intake of 4-12 year old Victorian children is 2369 mg/d [31], hence 505 mg sodium reduction is equivalent to a 21% reduction in daily intake.
