# Supplementary material for: The Development of a Web-Based Program to Reduce Dietary Salt Intake in Schoolchildren: Study Protocol
Source: JMIR Res Protoc. 2017 May 31;6(5):e103. doi: 10.2196/resprot.7597 (PMC5471360; doi:10.2196/resprot.7597)
Supplement: Multimedia Appendix 2 [file resprot_v6i5e103_app2.pdf]

Multimedia appendix 2. Sodium intake in Australian schoolchildren aged 6-16 years by eating occasion (n=2,921)[1]

**Meal time**	**% Daily Energy intake**		**Sodium (mg/d)**		**% Daily Sodium Intake**		**Sodium density (mg/1000 kJ)**	
	**mean**	**SD**	**mean**	**SD**	**mean**	**SD**	**mean**	**SD**
Breakfast 05:00-09:00	15.9	11.3	353	363	14.0	12.8	214	185
Morning tea 09:01-11:29	11.4	11.0	264	394	10.2	13.7	200	616
Lunch 11:30-14:00	19.8	13.4	689	662	25.9	19.1	363	374
Afternoon tea 14:01-16:59	13.8	13.3	334	528	12.3	15.7	223	737
Dinner 17:00-20:30	36.1	15.2	976	792	35.4	19.6	306	264
Supper 20:31-23:59	3.0	8.5	77	340	2.4	8.6	55	542

[1] Based on one day of 24-hr dietary recall data collected in the 2007 Children's Nutrition and Physical Activity Survey [27].
