# Supplementary material for: The Development of a Web-Based Program to Reduce Dietary Salt Intake in Schoolchildren: Study Protocol
Source: JMIR Res Protoc. 2017 May 31;6(5):e103. doi: 10.2196/resprot.7597 (PMC5471360; doi:10.2196/resprot.7597)
Supplement: Multimedia Appendix 3 [file resprot_v6i5e103_app3.pdf]

Multimedia appendix 3. Overview of intervention objectives, content and strategies mapped to behaviour change techniques/theory construct

Target	Objectives	Content and where relevant mapped to BCT/SCT theory construct
**Week 1. Session name: 'Salty Business'**		
**Child**	➢ Understand the relationship between sodium and salt	**Online session:** Relationship between salt and sodium presented by scientist storyline character. Video of sodium exploding in lake to engage child.
	➢ Understand the human body's requirement for sodium	**Online session:** Physiological requirement for salt intake and functions of salt within the body presented by doctor storyline character.
	➢ Be able to identify the recommended intake for sodium	**Online session:** Current salt intake of Australian children compared to dietary recommendations presented by nutritionist storyline character.
	➢ Understand the relationship between salt and cardiovascular health	**Online session:** Relationship between salt and cardiovascular health i.e. blood pressure and stress on heart) presented by nutritionist storyline character. (***5.1 Information about health consequences, SCT: knowledge)***
	➢ Engage child with storyline to complete 1st online session and sign up to the family challenge of eating no more than 5 g/day of salt per person	**Online session:** Comic strips to introduce background story and child signs up to be deputy detective.<br>**Online session:** Fun historical salt facts interactive activity delivered by historian storyline character.<br>**Online session:** On completion of the online session child is awarded 'Case File One' badge. ***(SCT: reinforcements)***
		**Online session:** To encourage children to think about where salt comes from in their diet they will be instructed to monitor their food intake for 1 meal of their choice (breakfast, lunch, dinner) during week 1.
**Parent**	➢ Understand the relationship between sodium and salt	**Online newsletter:** Relationship between salt and sodium.<br>**Website:** Handout for conversion of sodium to salt.
	➢ Understand the human body's requirement for sodium	**Online newsletter:** Physiological requirement for salt intake and functions of salt within the body.
	➢ Be able to identify the recommended intake for sodium	**Online newsletter:** Current salt intake of Australian children and adults compared to dietary recommendations.
	➢ Understand the relationship between salt and health outcomes	**Online newsletter and link to scribe video:** Information on health outcomes associated with high salt intakes in children and adults provided (e.g. high blood pressure, tracking of blood pressure across life course, kidney disease, osteoporosis, stomach cancer, development of taste preferences for salty foods). (***5.1 Information about health consequences)***<br>**SMS:** DELISH: Cutting down on salt, can help keep our blood pressure levels healthy and protect our hearts. (***5.1 Information about health consequences)***
**Week 2. Session name: 'Hidden & Visible Salt'**		
**Child**	➢ Understand that most salt comes from processed foods, less comes from discretionary salt and unprocessed foods do not contain added salt	**Online session:** Via interactive session, child visits processed food factory and farm and uses 'salt detector' to test different foods at each site to learn what foods have added salt ***(SCT: self-efficacy)***<br><br>**Online session:** The contribution of salt from the salt shaker is explained by farmer storyline character.
	➢ Be able to identify foods with added salt	**Online session:** Interactive game 'Find the Hidden Salt' where children are presented with commonly consumed foods across breakfast, lunch and dinner and asked to identify which foods contain added salt. (***8.1 Behavioural practice/rehearsal, SCT: self-efficacy)***

	➢ Key behavioural message introduced 'STOP using the salt shaker'	**Online session:** Facilitated goal setting with child (i.e. child picks preferred goal from list of options related to limiting use of table salt). ***(1.1 Goal setting behaviour, SCT: intentions)***<br><br>**Online session:** Interactive activity whereby child is prompted to identify potential barriers to limiting use of table salt ***(1.2 Problem solving)***<br><br>**Starter Pack:** Fun 'STOP – don't use the salt shaker' sticker to place on salt shaker at home. ***(7.1 Prompts/cues)***
		**Online session:** On completion of the online session child is awarded 'Case File Two' badge. ***(SCT: reinforcements)***
**Parent**	➢ Understand that most salt comes from processed foods, less comes from discretionary salt and unprocessed foods do not contain added salt.	**Online newsletter:** Information on sources of salt.
	➢ Be able to identify the major contributors of salt to children's diets	**Online newsletter:** Top sources of salt in children's diets.
	➢ Key behavioural message introduced 'STOP using the salt shaker'	**Starter Pack:** Fun 'STOP – don't use the salt shaker' sticker to place on salt shaker at home. ***(7.1 Prompts/cues)***<br><br>**Online newsletter and website:** Resource for using herbs and spices to flavour food instead of salt. ***(4.1 Instruction on how to perform the behaviour)***<br><br>**Online newsletter:** Relationship between salt intake and taste preferences for salt in food.
**Week 3. Session name: 'Sneaky Salt'**		
**Child**	➢ Mini-challenge check in to review progress on previous week's goal	**Online session:** Child will be asked to report on progress on meeting week 2 goal. If child reports changing the behaviour bonus badge awarded. ***(10.10 Reward (outcome), SCT: reinforcements)***
	➢ Be familiar with the Australian Guide to Healthy Eating (AGTHE) and be able to identify 'core' foods that fall within the five food groups	**Online session:** Australian Guide to Healthy Eating model introduced and the role of the five food groups in a healthy diet is presented by nutritionist storyline character.<br>**Starter pack:** Fridge magnet of AGTHE provided.
	➢ Understand that some 'core' foods contribute salt to the diet	**Online session:** Core foods without added salt are coined as 'Salt Free Champions'. Core foods with added salt are coined as 'Sneaky Salties'. Contribution of salt from bread, breakfast cereal and cheese to diet is presented by nutritionist storyline character.
	➢ Interpret sodium information included on the nutrition information panel (NIP) on food labels	**Online session:** Child specific video clip providing instruction on how to interpret sodium information on food labels and select foods with less salt. ***(4.1 Instruction on how to perform the behaviour)***<br><br>**Website:** Handout including practice exercises using NIP to pick foods with less salt ***(SCT: self-efficacy)***
	➢ Be able to use the NIP on food labels to pick foods with less salt	**Online session:** Interactive activity where child views food products within a supermarket and is asked to use the information on food labels of paired product items to select the product with less salt. ***(8.1 Behavioural practice/rehearsal, SCT: self-efficacy, behavioural capability)***
	➢ Key message introduced 'SWITCH to lower salt foods by checking food	**Online session:** Facilitated goal setting with child to switch to a lower salt food item (e.g. bread, breakfast cereal and/or cheese). ***(1.1 Goal setting behaviour, SCT: intentions)***

	labels' and child sets related goal	**Online session:** Interactive activity whereby child is prompted to identify potential barriers to switching to lower salt foods and strategies to overcome them. ***(1.2 Problem solving)***
		**Online session:** On completion of the online session child is awarded 'Case File Three' badge. ***(SCT: reinforcements)***
**Parent**	➢ Be familiar with the Australian Guide to Healthy Eating (AGTHE) and be able to identify 'core' foods that fall within the five food groups	**Online newsletter:** Information on AGTHE and five food groups provided.<br>**Starter pack:** Fridge magnet of AGTHE provided + Eat for Health education brochures.
	➢ Understand that some 'core' foods contribute to salt intake	**Online newsletter:** Contribution of salt from bread, cheese and breakfast cereal to children's salt intake.
	➢ Interpret sodium information included on the nutrition information panel (NIP) on food labels	**Online newsletter:** Information on reading food labels and sodium information provided, examples included.
	➢ Be able to use the NIP on food labels to pick foods with less salt	**Video (link included in online newsletter):** Parent specific video clip providing instruction on how to interpret sodium information on food labels and select foods with less salt **(6.1 Demonstration of the behaviour)**
	➢ Interpret sodium/salt information included on front of pack food labels	**Online newsletter:** Information on interpreting reduced salt and no added salt claims on front of pack and what products to look for these on.
	➢ Key message introduced 'SWITCH to lower salt foods by checking food labels'	**Smartphone Application:** Via online newsletter parent is directed to download freely available FoodSwitch App (Bupa & The George Institute for Health) which can be used to find lower salt food products. Information brochure for App also included in Starter Pack.<br><br>**Website:** Recipes for lower salt meals (breakfast, lunch, dinner, and snack) provided, highlighting the amount of salt that can be saved with recipe modification. ***(4.1 Instruction on how to perform the behaviour)***<br><br>**Website:** Top picks handout detailing lower salt brands for bread, breakfast cereal and cheese. ***(4.1 Instruction on how to perform the behaviour)***
**Week 4. Session name: 'Salt Swaps'**		
**Child**	➢ Mini-challenge check in to review progress on previous week's goal.	**Online session:** Child will be asked to report on progress on meeting week 3 goal. If child reports changing the behaviour bonus badge awarded. ***(10.10 Reward (outcome), SCT: reinforcements)***
	➢ Be able to identify 'discretionary' foods and their role in the diet.	**Online session:** Within the context of the AGTHE 'discretionary' foods are introduced by nutritionist storyline character.<br>**Online session:** Interactive activity where child is asked to select 'discretionary' foods found within a kitchen.
	➢ Understand the contribution of discretionary foods to salt intake.	**Online session:** Discretionary foods that contain added salt are coined as 'Salt Offenders'. Contribution of salt from discretionary foods e.g. take-away hamburgers/pizza, meat pies is presented by nutritionist storyline character.
	➢ Be able to identify 'discretionary' foods that contain added salt	**Online session:** Interactive activity where child is asked to select 'discretionary' foods that contain added salt.
	➢ Be able to identify salt free 'core' foods that could replace 'discretionary' foods in the diet.	**Online session:** Interactive Kitchen Quest game where child views different meals and is asked to identify 'Salt Offender' and replace them with an appropriate 'Salt Free Champion'. ***(8.1 Behavioural practice/rehearsal)***

	➢ Key message introduced 'SWAP processed salty foods with healthier alternatives'	**Online session:** Facilitated goal setting with child to select one processed salty food (i.e. Salt Offender) currently included in diet that can be swapped to a healthier alternative (i.e. Salt Free Champion) ***(1.1 Goal setting behaviour, SCT: intentions)***<br><br>**Online session:** Interactive activity whereby child is prompted to identify potential barriers to swapping to healthier alternative **(1.2 Problem solving)**<br><br>**Website:** Handout identifying commonly consumed processed salty foods and suggested healthier alternative. ***(4.1 Instruction on how to perform the behaviour)***
		**Online session:** On completion of the online session child is awarded 'Case File Four' badge. ***(SCT: reinforcements)***
**Parent**	➢ Be able to identify 'discretionary' foods and their role in the diet.	**Online newsletter:** Discretionary foods within the context of AGTHE.
	➢ Understand the contribution of discretionary foods to salt intake.	**Online newsletter:** Contribution of commonly consumed discretionary foods to salt intake.
	➢ Be able to identify salt free 'core' foods that could replace 'discretionary' foods in the diet.	**Online newsletter:** Example lunch box, dinner and snack ideas provided, with targeted foods to swap to reduce the amount of salt in the diet. ***(4.1 Instruction on how to perform the behaviour)***
	➢ Key message introduced 'SWAP processed salty foods with healthier alternatives'	**Website:** Recipes for lower salt condiments/savoury sauces provided, highlighting the amount of salt that can be saved with recipe modification. ***(4.1 Instruction on how to perform the behaviour)***
		**Online newsletter:** Common salt myths debunked.
**Week 5. Wrap-up session**		
**Child**	➢ Mini-challenge check in to review progress on previous week's goal.	**Online session:** Child will be asked to report on progress on meeting week 4 goal. If child reports changing the behaviour bonus badge awarded. ***(10.10 Reward (outcome), SCT: reinforcements)***
	➢ Understand key behaviours to reduce salt in the diet	**Online session:** Interactive quiz to review main content and key behavioural messages of the program.
		**Online session:** On completion of final online session the child is promoted to a chief investigator and hard copy certificate for completion of the program is provided. (***SCT: reinforcements)***
**Parent**	➢ Understand key behaviours to reduce salt in the diet	**Online newsletter:** Key content and behavioural messages reviewed.
