# Supplementary material for: The Development of a Web-Based Program to Reduce Dietary Salt Intake in Schoolchildren: Study Protocol
Source: JMIR Res Protoc. 2017 May 31;6(5):e103. doi: 10.2196/resprot.7597 (PMC5471360; doi:10.2196/resprot.7597)

Multimedia appendix 4. Overview of format for weekly online sessions for children

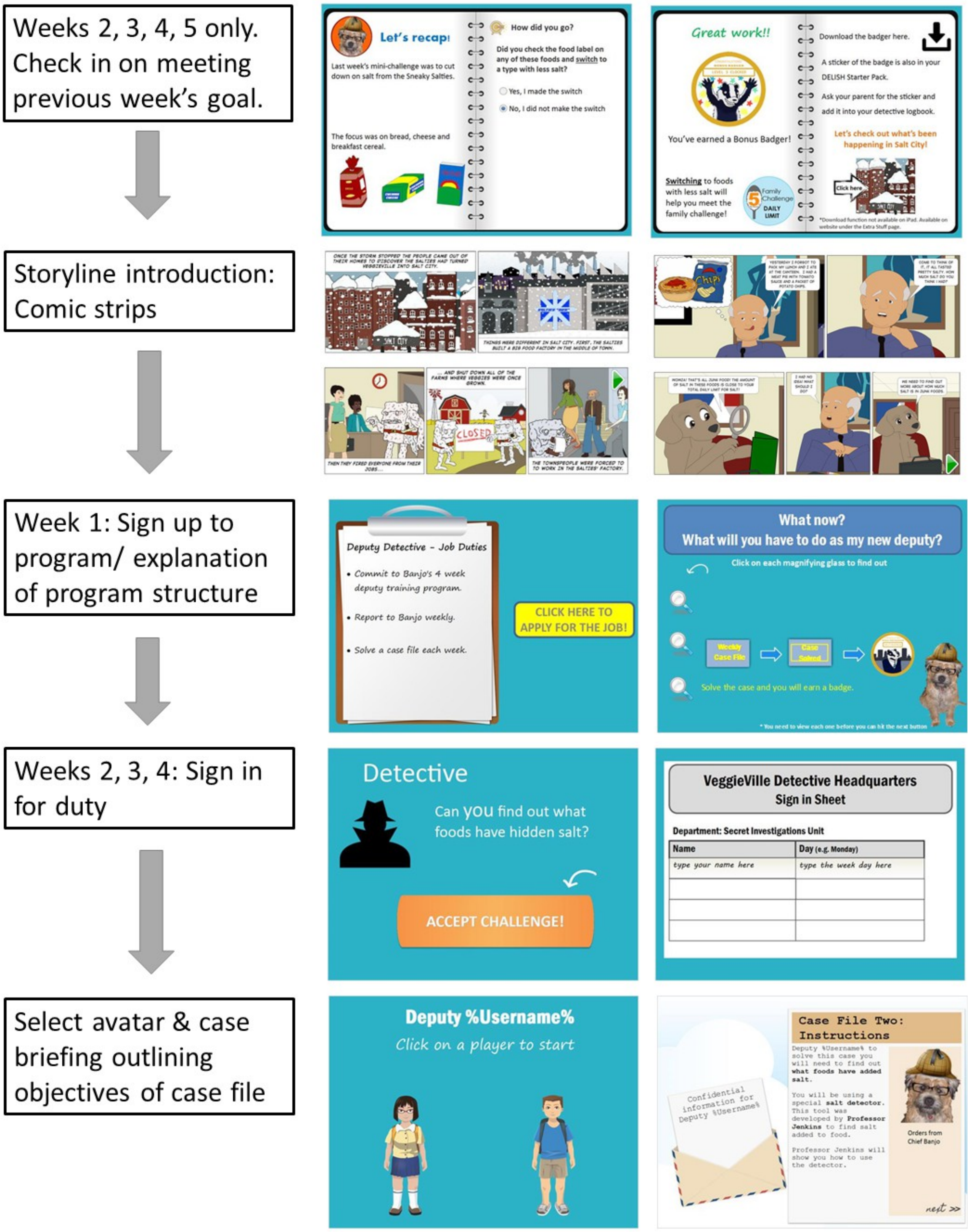

Content delivered via characters embedded within the case file.

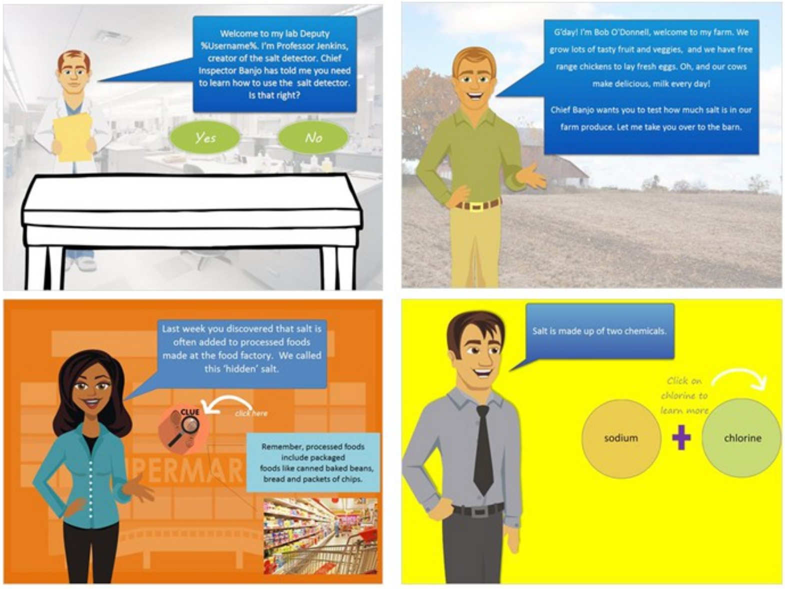

Interactive activities to enhance learning & practice behaviors e.g. reading food labels, testing to find out what foods have added salt.

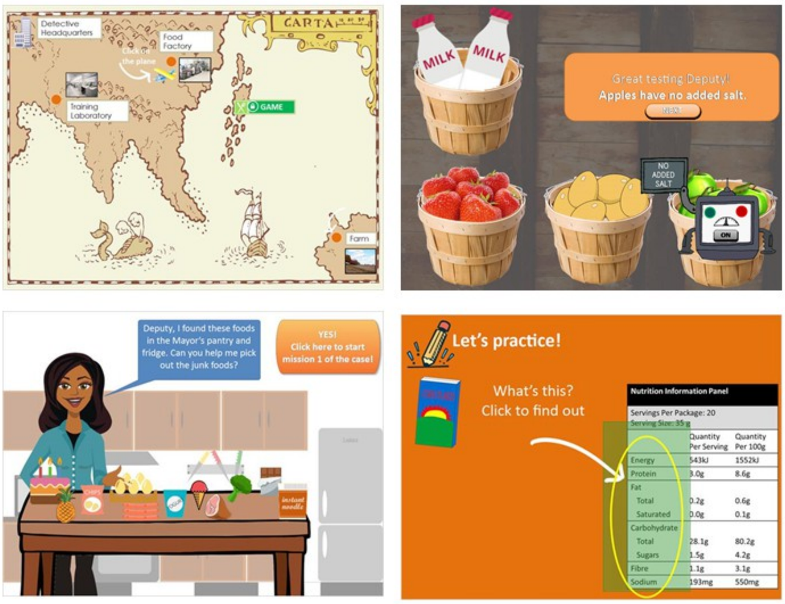

Challenge to solve case file, presented in game format.

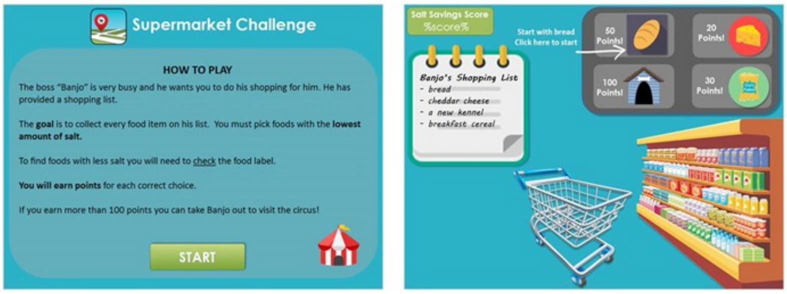

Case file solved, badge provided.

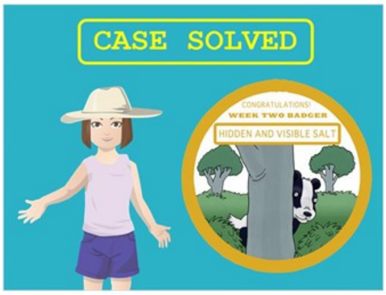

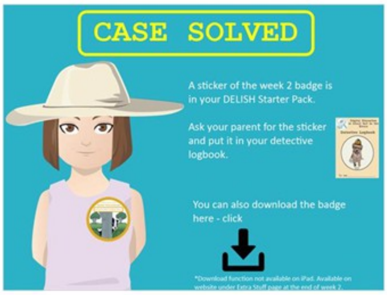

Recap on key points from this week's case.

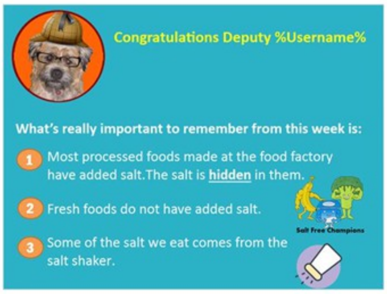

Mini-challenge:
Child sets weekly goal

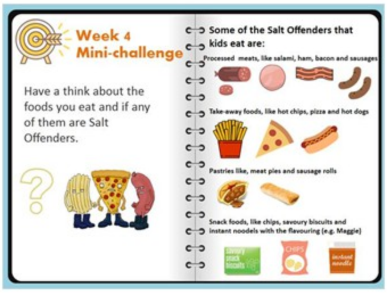

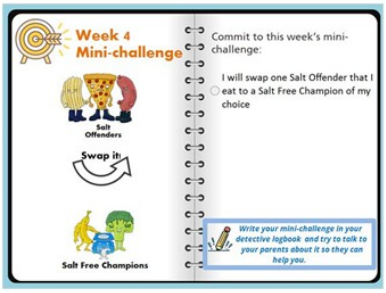

Mini-challenge:
Possible barriers and solutions to meeting goal.

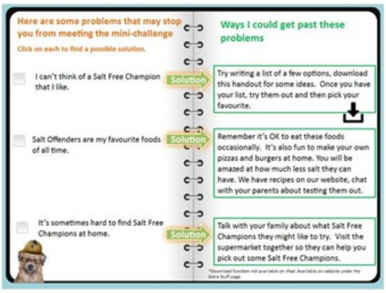

Sign off page/
Case file solved
*Reminder that resources can be accessed via study website

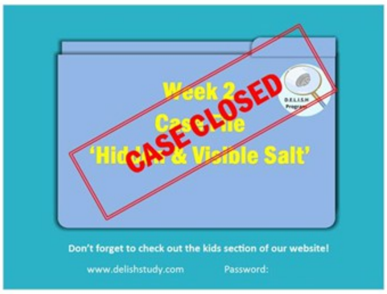

Supplement: Multimedia Appendix 4 [file resprot_v6i5e103_app4.pdf]
