# Supplementary material for: The Development of a Web-Based Program to Reduce Dietary Salt Intake in Schoolchildren: Study Protocol
Source: JMIR Res Protoc. 2017 May 31;6(5):e103. doi: 10.2196/resprot.7597 (PMC5471360; doi:10.2196/resprot.7597)
Supplement: Multimedia Appendix 5 [file resprot_v6i5e103_app5.pdf]

Multimedia appendix 5. Selection of quotes from interviews during pilot testing of two online sessions

“The instructions of session one were easy to understand, I thought the badgers were really funny.”
“I would like a copy of the badger as a sticker”
“Maybe one or two more avatars to select would be good”
“The salt detector made sense to me. There were a few too many foods to test with the salt detector, it would be better with maybe two less foods”
"I liked the salt detector but when I was using it on the food conveyor belt I thought they were moving too fast. I would get them on the second try"
“'I thought the salties looked funny, it was funny that the salties were very muscly”
“The information before the game helped me to complete the game and answer the questions”
"I liked going around and interviewing the scientist and stuff" "I liked finding out about information in different ways, in the first session interviews and in the second session using the food tester"
“I could read all of the words but some I had to ask mum what they meant”
"I read all of the information [in session 1] but I thought it could have been a bit shorter. Maybe less information with the doctor"
“Sometimes the text was too fast and quick to disappear”
"The dog was great, I actually really liked the dog the detective dog. The storyline was pretty good and I liked the salties"
“The comics were cool”
“I liked the little mini games and the map at session one. I think one area to improve is the avatars. If you get enough credits/energy you could customize your avatars to change the look of them”
